# In vitro characteristics of *Lactobacillus* spp. strains isolated from the chicken digestive tract and their role in the inhibition of *Campylobacter* colonization

**DOI:** 10.1002/mbo3.512

**Published:** 2017-07-24

**Authors:** Patrycja A. Kobierecka, Agnieszka K. Wyszyńska, Tamara Aleksandrzak‐Piekarczyk, Maciej Kuczkowski, Anna Tuzimek, Wioletta Piotrowska, Adrian Górecki, Iwona Adamska, Alina Wieliczko, Jacek Bardowski, Elżbieta K. Jagusztyn‐Krynicka

**Affiliations:** ^1^ Faculty of Biology Department of Bacterial Genetics Institute of Microbiology University of Warsaw Warsaw Poland; ^2^ Polish Academy of Sciences Institute of Biochemistry and Biophysics Warsaw Poland; ^3^ Faculty of Veterinary Medicine Department of Epizootiology and the Clinic of Birds and Exotic Animals University of Environmental and Life Sciences Wrocław Poland; ^4^ Faculty of Biology Department of Animal Physiology Institute of Zoology University of Warsaw Warsaw Poland

**Keywords:** *Campylobacter*, *Lactobacillus*, poultry, probiotics

## Abstract

*Campylobacter jejuni*/*coli* infections are the leading cause of bacterial diarrheal illnesses in humans. Many epidemiological studies indicate that improperly prepared meat from chickens that carry a high load of *Campylobacter* in their intestinal tracts is the key source of human infections. LAB, mainly members of the *Lactococcus* and *Lactobacillus* genera, increasingly have been tested as vehicles for the delivery of heterologous bacterial or viral antigens to animal mucosal immune systems. Thus, the objective of this study was to isolate, identify, and characterize *Lactobacillus* spp. strains isolated from chickens bred in Poland. Their ability to decrease the level of bird gut colonization by *C. jejuni* strain was also analyzed. First, the influence of the different chicken rearing systems was evaluated, especially the effect of diets on the *Lactobacillus* species that colonize the gut of chickens. Next, selected strains were analyzed in terms of their anti‐*Campylobacter* activity in vitro; potential probiotic traits such as adhesion properties, bile and low pH tolerance; and their ability to grow on a defined carbon source. Given that improperly prepared chicken meat is the main source of human infection by *Campylobacter,* the selected strains were also assessed for their ability to inhibit *Campylobacter* colonization in the bird's intestine. These experiments revealed enormous physiological diversity among the *Lactobacillus* genus strains. Altogether, our results showed that *L. plantarum* strains isolated from the digestive tracts of chickens bred in Poland displayed some probiotic attributes in vitro and were able to decrease the level of bird gut colonization by *C. jejuni* strain. This suggests that they can be employed as vectors to deliver *Campylobacter* immunodominant proteins to the bird's immune system to strengthen the efficacy of in ovo vaccination.

## 1 INTRODUCTION

Microbiota present in human or animal intestinal tracts have enormous impact on the health of the host. Recent advances in technology, in particular progress made in the field of DNA sequencing combined with many bioinformatics methods has replaced culture‐dependent methods with culture‐independent strategies. Data from such analyses reveal details of microbiota diversity and their interactions with the host. Microbial communities of the chicken gastrointestinal tract (GIT), as well as the chicken microbiome, have recently been analyzed by many research group to better understand their influence on bird health and improve poultry industry productivity (for review see [Oakley, Lillehoj, et al., 2014]). The diverse chicken microbiota consists of over 900 bacterial species (Wei, Morrison, & Yu, 2013). Although the microbial communities found in different sections of the chicken GIT vary and their members belong to 13 phyla, the most dominant phyla are Firmicutes, Bacteroides, and Proteobacteria (Pan & Yu, 2014; Stanley, Hughes, & Moore, 2014). Kaakoush et al. (2014), who studied the microbiota of chickens from two farms, assigned them to four enterotypes dominated by varies phyla. Many factors influence the composition of the chicken microbiota; the most substantial factors are the chicken breeds, bird age, and farming practices (Kaakoush et al., 2014; Oakley, Buhr, et al., 2014; Schokker et al., 2015; Thibodeau et al., 2015).

Campylobacteriosis is the most frequently reported foodborne zoonosis worldwide (Kaakoush, Castano‐Rodriguez, Mitchell, & Man, 2015). As chickens harbor a high load of *Campylobacter jejuni* in their GIT, improperly prepared chicken meat is the main source of human campylobacteriosis (Agunos, Waddell, Leger, & Taboada, 2014; Hermans et al., 2012; Kaakoush et al., 2015; Zambrano, Levy, Menezes, & Freeman, 2014). To date, many interventions to combat the chicken colonization by *C. jejuni*, such as introducing different hygiene barriers at farms, various nutritional strategies or using bacteriophages, have been examined with varying success (Meunier, Guyard‐Nicodeme, Dory, & Chemaly, 2016; Newell et al., 2011; Wagenaar, French, & Havelaar, 2013). Given that members of the *Lactobacillus* genus are predominant in the chicken microbiota and that some of them can be classified as probiotics, their anti‐*Campylobacter* activities have recently been intensively examined using different strategies (Chaveerach, Lipman, & van Knapen, 2004; Robyn, Rasschaert, Messens, Pasmans, & Heyndrickx, 2012; Santini et al., 2010). The aim of these studies was to manipulate the chicken microbiota in order to inhibit chicken digestive tract colonization by *Campylobacter* (Ghareeb et al., 2012; Santini et al., 2010; Willis & Reid, 2008). The studies showed that only a small percentage of *Lactobacillus* strains isolated from chicken intestinal tracts or from fecal samples are able to compete with *Campylobacter*. Although a lot of *Lactobacillus* isolates have demonstrated high anti‐*Campylobacter* activity in vitro*,* many only slightly reduce bird colonization by the pathogen when administered in vivo, which demonstrates the complex mechanisms of interactions among microbiota constituents (Chaveerach et al., 2004; Robyn et al., 2012; Santini et al., 2010). *C. jejuni* genetic diversity makes the issue even more complicated as inhibition of the pathogen's growth by a specific *Lactobacillus* isolate is dependent on the *C. jejuni* strain used in the experiment (Chaveerach et al., 2004; Santini et al., 2010). In some cases, probiotic formulations composed of several bacteria, even belonging to different genera, were proven to be the most effective (Ghareeb et al., 2012; Willis & Reid, 2008). The mechanisms by which *Lactobacillus* isolates inhibit *Campylobacter* growth in vivo are diverse and not fully understood. The source of the *Lactobacillus* strain appears not to be a critical factor, though the data are sometimes contradictory (Chaveerach et al., 2004; Lin, Yu, Jang, & Tsen, 2007; Nishiyama et al., 2014; Santini et al., 2010). Several mechanisms that potentially depend on both the pathogen and the probiotic may be involved in the competitive inhibition of *C. jejuni* by *Lactobacillus*. Those that have been proven to play a significant role in inhibition include adhesion to the intestinal epithelium of the host (Nishiyama et al., 2014; Spivey, Dunn‐Horrocks, & Duong, 2014); coaggregation and autoaggregation (Tareb, Bernardeau, Gueguen, & Vernoux, 2013); production of antimicrobial compounds, such as organic acid, bacteriocin (Messaoudi et al., 2013; Neal‐McKinney et al., 2012; Svetoch & Stern, 2010), and hydrogen peroxide (Mota et al., 2006); and immuno‐modulatory effects (Messaoudi et al., 2012).

The objective of this study was to isolate, identify, and characterize the *Lactobacillus* spp. strains that constitute the chicken intestinal tract microbiota. The first aim of the study was to evaluate the influence of different rearing systems, especially, the dietary treatment of chickens, on *Lactobacillus* species colonizing their gut. Next, the selected strains were analyzed in terms of their anti‐*Campylobacter* activity in vitro and their ability to colonize chicken's gut (adhesion properties, bile and low pH tolerance). Their metabolic properties, ability to grow on a defined carbon source, were also evaluated. Given that improperly prepared chicken meat is the main source of human infection by *Campylobacter*, the ability of the selected strains to inhibit colonization of the bird's intestine by the pathogen was also examined.

## 2 MATERIALS AND METHODS

### 2.1 Bacterial strains media and growth conditions

Bacterial strains used in this study are listed in Table 1. A total of 107 *Lactobacillus* strains were isolated from privately owned “back‐yard” flocks (64 strains) and commercial broiler chicken flocks (43 strains) in Poland. *Lactobacillus* strains were cultured in MRS broth (de Man, Rogosa, and Sharpe) or MRS agar (solidified with 1.5% agar) medium (Oxoid Ltd., Hampshire, England) at 37°C under microaerobic conditions (85% N_2_, 10% CO_2_, 5% O_2_).

**Table 1:** Bacterial strains used in this study

Strain	Relevant characteristics	Source or reference
*C. jejuni* 81–176	Human clinical isolate	Korlath et al. (1985)
*C. jejuni* 12	Chicken isolate	Wyszynska et al. (2004)
*L. salivarius* PA14C	Chicken isolate	This study
*L. salivarius* PA17D	Chicken isolate	This study
*L. salivarius* PA18C	Chicken isolate	This study
*L. reuteri* PA12C	Chicken isolate	This study
*L. reuteri* PA8A	Chicken isolate	This study
*L. reuteri* PA19B	Chicken isolate	This study
*L. crispatus* PA11D	Chicken isolate	This study
*L. plantarum* PA11A	Chicken isolate	This study
*L. plantarum* PA18A	Chicken isolate	This study
*L. plantarum* PA20A	Chicken isolate	This study
*L. agilis* PA16B	Chicken isolate	This study

*C. jejuni* 12 (PCM 2852, Polish Collection of Microorganisms) and *C. jejuni* 81–176 (ATCC^®^ BAA‐2151, American Type Culture Collection, USA) were used in *C. jejuni* inhibition assays in vitro. The *C. jejuni* 12/2 strain employed in the protection experiment was a broiler‐isolated strain labeled with the pUOA18 plasmid containing a *cat* gene. Previous experiments have shown that the pUOA18 plasmid is stably maintained in *Campylobacter* (Wyszynska, Raczko, Lis, & Jagusztyn‐Krynicka, 2004). *Campylobacter* strains were cultured under microaerobic conditions in Blood Agar (BA) (Oxoid Ltd.) plates supplemented with 5% horse blood and *Campylobacter* Selective Supplement (Oxoid Ltd. at 37°C. The medium was supplemented with chloramphenicol (15 μg/ml), if necessary.

### 2.2 Isolation and identification of lactic acid bacteria

Twenty stool samples from back‐yard chickens or cloacal swabs from 25 commercial broiler chickens were streaked onto selective MRS agar and incubated overnight in a microaerobic atmosphere at 37°C. After 24 hr incubation, colonies were randomly picked from the plates and subcultured two or three times on fresh MRS agar plates. The pure LAB cultures were then kept in MRS broth supplemented with 30% (v/v) glycerol and frozen at −80°C until further analysis.

Preliminary identification consisted of Gram staining to assess the morphology of cells by optical microscopy. Next, amplification of the 16S rDNA gene was performed for all gram positive strains. For this, chromosomal DNA was isolated from overnight cultures of selected strains. Pellets were resuspended in TES (25 mmol/L sucrose, 50 mmol/L Tris HCl pH 8.0, 10 mmol/L EDTA) containing lysozyme (20 mg/ml, Sigma) and mutanolysin (500 u/ml, A&A Biotechnology). After incubation for 1 hr at 37°C under constant shaking, chromosomal DNA from the bacterial strains was isolated using a commercial kit (A&A Biotechnology, Poland). Polymerase chain reactions (PCR) were performed with Color optiTaq (EURX) or HotStar HiFidelity Polymerase (Qiagen) under standard conditions. For amplification of the 16S rDNA gene, universal primers F27 (5′‐AGAGTTTGATCCTGGCTCAG‐3′) and 1492 (5′‐GGTTACCTTGTTACGACTT‐3′) were used (McDonald, Kenna, & Murrell, 1995) with an expected PCR product size of 1.5 kb. PCR products were purified using the Clean‐up Concentrator kit (A&A Biotechnology, Poland). Synthetic oligonucleotide synthesis and DNA sequencing of the PCR product was performed by Genomed S.A. (Warsaw, Poland), and then the nucleotide sequences were analyzed using BLAST against the nucleotide database at the NCBI website.

### 2.3 *C. jejuni* inhibition assays in vitro

The *C. jejuni* inhibition assay was performed on BA plates containing 5% horse blood (Oxoid) inoculated with 100 μl of *Campylobacter* cultures (*C. jejuni* 12 or *C. jejuni* 81–176), prepared by suspending the microorganisms in 0.9% NaCl and adjusting the density to 0.5 McFarland standard (~1 × 10^8 ^CFU/ml). Plates were incubated for 2 hr at 37°C under microaerobic conditions. Overnight cultures of lactobacilli were centrifuged, and to determine if bacteriocins contributed to inhibition, part of supernatant was neutralized to neutral pH with 6 N NaOH. Untreated supernatants and supernatants neutralized to pH 7 were subsequently filter sterilized (0.22 μm) and spotted onto MRS agar plates inoculated with *Campylobacter*. Plates were incubated for 24–48 hr at 37°C under microaerobic conditions. Growth inhibition was evaluated as inhibition around the spotted *Lactobacillus* supernatant.

### 2.4 Detection of lactic acid production

Detection of lactic acid was carried out as described earlier by Neal‐McKinney et al. (2012). The concentrations of d‐ and l‐lactate in the cell‐free supernatants were measured using stereo‐specific d‐ and l‐lactate assay kits (Megazyme, Wicklow, Ireland). The measurements for selected strains were performed in triplicate for reproducibility. Assays were performed with pure solutions of d‐ and l‐lactate to ensure that the kits were stereo‐specific.

### 2.5 Tolerance to acid and bile

Tolerance to low pH and bile content was assessed as described by Bujnakova et al. (Bujnakova, Strakova, & Kmet, 2014), with minor modifications. Briefly, 100 μl lactobacilli from overnight cultures (37°C, MRS broth, 5% CO_2_) diluted to OD_660 nm_ of 1.0 was inoculated into 10 ml of following test solutions: MRS broth control, MRS broth adjusted to pH 2.5, and MRS broth containing 0.5% oxgall (Difco). As a control, broth without inoculation was used. For survival under the different conditions, samples were taken at 6 hr and 100 μl cultures were plated on MRS agar after appropriate dilution. Colonies were counted after 48 hr incubation at 37°C. The survival rate was calculated as the log_10_ value of CFU/ml.

### 2.6 Resistance to sodium chloride

To assess effects of osmolarity, 3 μl of o/n culture of each *Lactobacillus* strain was transferred onto plates with MRS agar (Merck, Germany) and MRS agar containing: 0.34 mol/L (2%), 0.69 mol/L (4%), 1 mol/L (6%), or 1.36 mol/L (8%) (w/v) NaCl (Sigma, USA) and incubated at 37°C for 48 hr. The survival of bacteria was examined visually by comparison of their growth efficiency on MRS agar with their growth on MRS agar with different concentrations of NaCl. Each strain was tested in two independent experiments.

### 2.7 Sugar fermentation pattern

To determine sugar fermentation profiles of the *Lactobacillus* spp., API 50 CH kit was used according to the manufacturer's instructions (BioMerieux, France). The resulting fermentation pattern was inspected following anaerobic incubation at 37°C after 48 hr. Fermentation of a carbohydrate was detected by acid production, as demonstrated by a color change in the pH indicator present in the medium. Each strain was tested in three independent experiments.

### 2.8 Adhesion to bare polystyrene

Adhesion of bacterial cells to polystyrene (PS) was tested on the 96‐well microtiter PS plates (Thermo Fischer Scientific Nunc A/S, Denmark) using a modification of the method already described (Radziwill‐Bienkowska, Zochowska, Bardowski, Mercier‐Bonin, & Kowalczyk, 2014). Briefly, bacteria from overnight cultures diluted to OD_660 nm_ of 1.0 were harvested by centrifugation (10^3^ *g*; 1 min) and resuspended in an equal volume of PBS (BioShop, Canada). A volume of 100 μl of bacterial suspension was added to each well (eight for each strain). To remove unbound bacteria after 3 hr incubation under static conditions at 37°C, the wells were washed three times with 200 μl of sterile MilliQ‐grade water. Bound cells were stained with crystal violet (Scharlau, Spain) (100 μl per well, RT; 10 min), and excess stain was removed by rinsing three times with water, as above. Finally, stained bacteria were suspended in 200 μl of 96% ethanol and optical density was determined at 583 nm on a Synergy HT Multi‐Detection Reader (BioTek Instruments Inc., USA). The average value of at least six measurements was calculated after rejecting extreme results. Bacterial adhesion was determined in three independent experiments and the results are presented as mean ± *SD*. Each microtiter plate included blank wells with PBS and control GI (gastrointestinal) strains: high‐adhesive *L. rhamnosus* GG (Segers & Lebeer, 2014) and nonadhesive *L. rhamnosus* LOCK 0908 (Aleksandrzak‐Piekarczyk et al., 2016).

To calculate the adherence ratio of bacteria to PS, the value of absorbance of bacteria was divided by the absorbance of the control sample (PBS, only). Bacteria were characterized as strongly adherent (*A* ≥ 6), moderately adherent (6 > *A* ≥ 3), weakly adherent (3 > *A *> 2), and nonadherent (*A *≤ 2).

### 2.9 *Campylobacter* colonization experiment

Experiments were performed on Hy‐line chickens hatched and reared under controlled conditions from the day of hatch. The chickens were kept under controlled light (L:D 12:12) and temperature (32 ± 2°C during first week and 24 ± 2°C thereafter) conditions, with free access to the standard food and water. Chickens were confirmed to be culture‐negative for *Campylobacter* by cloacal swabbing. All animal experiments were carried out according to the ethical standards and approval (No. 743/2015) of the Local Ethics Committee No. 1, Warsaw, Poland.

Birds were randomly assigned to six experimental groups (each containing 12 individuals) and housed in an animal facility, with separate cages for each group. On the day of hatch and 4 days post hatch, all groups of chicks were inoculated by oral gavage with 0.1 ml lactobacilli suspension (~10^8^ CFU/ml): *L. reuteri* PA8A, *L. crispatus* PA11D, *L. salivarius* PA14C, *L. plantarum* PA18A, *L. plantarum* 20A. A group of birds inoculated with BSG (PBS with 0.1% gelatin) was used as a control. Following vaccination, chickens were observed for development of diarrhea and other potential adverse side effects. At the 14th day of life, birds were orally challenged with ~10^4^ CFU of *C. jejuni* wild‐type strain 12/2. At 4 and 8 days post challenge (22nd day of life), six birds from each group were killed and samples of cecum were collected. Dilutions of the contents were made in PBS and plated onto BA plates supplemented with 5% horse blood, “*Campylobacter* Selective Supplement (Blaser‐Wang)” and chloramphenicol (15 μg/ml) for enumeration of *C. jejuni*. Plates were incubated at 37°C for 48 hr. Plates that were culture‐negative at 48 hr were reincubated for an additional 48 hr. This procedure permits detection of 10^3^ CFU/g of cecal contents.

## 3 RESULTS

### 3.1 Isolation and identification of *Lactobacillus* strains

The *Lactobacillus* genus includes more than 200 species (http://www.ncbi.nlm.nih.gov/Taxonomy/Browser/wwwtax.cgi?id=1578, August 2016) that are characterized by diverse physiological properties (Bernardeau, Vernoux, Henri‐Dubernet, & Gueguen, 2008; Lukjancenko, Ussery, & Wassenaar, 2012).

Thus, in this study, we decided to search for *Lactobacillus* strains with potential probiotic activity among strains isolated from chickens breeding in Poland and we asked whether different chicken rearing systems, especially dietary treatment, have an impact on *Lactobacillus* species colonizing their gut (Bjerrum et al., 2006; Pan & Yu, 2014).

In total, 107 *Lactobacillus* strains were isolated: 64 from fecal samples of two privately owned “back‐yard” chicken farms and 43 from cloaca of birds reared in seven commercial poultry farms specializing in production of consumer eggs. The hens (Lohman Brown) from farms were at laying age, 22–50 weeks old. The chickens were fed a commercially available feed for laying hens that consists of raw materials (wheat, triticale, corn, chalk, vegetable oils, vitamins, amino acids, and microelements). This feed does not contain growth‐promoting antibiotics or coccidiostats.

Chicken stool samples were streaked onto selective MRS agar and incubated within an anaerobic atmosphere overnight at 37°C. Pure cultures were obtained. *Lactobacillus* genus membership was confirmed by molecular analysis (PCR amplification of the intergenic chromosomal DNA region between the 16S rDNA and 23S rDNA and evaluation of the sizes of the DNA fragments). To determine the species of the isolates, the 16S rDNA genes were amplified, and the PCR products were sequenced and analyzed using BLAST against the nucleotide database at the NCBI website. The strains were also characterized by morphological and microscopic observations (Figure S1).

**Figure 1:**
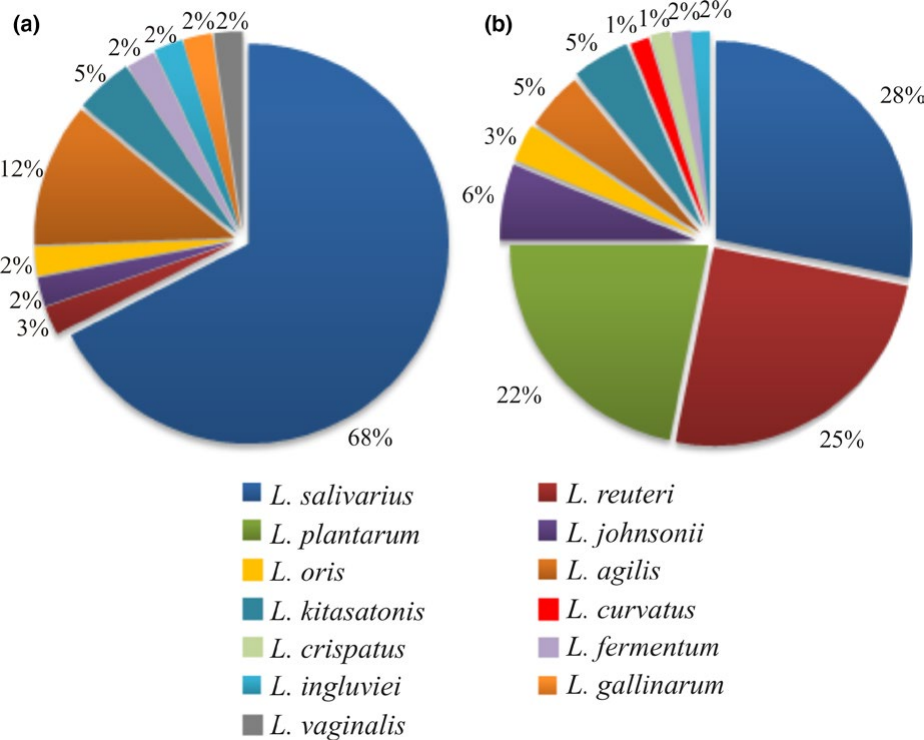
Pie charts represent relative abundance of *Lactobacillus* species isolated from cloaca of commercial broiler chickens (a) and from stools of backyard chickens (b)

Generally, the most abundant *Lactobacillus* species was *L. salivarius,* regardless of chicken breeding methods. *L. salivarius* (68%) and *L. agilis* (12%) were more prevalent than other species of *Lactobacillus* genus obtained from the farm chickens. The species distribution among all the *Lactobacillus* species isolated from fecal samples of “back‐yard” chickens was more diverse. *L. salivarius* (29%) was the dominant species, followed by *L. reuteri* (24%) and *L. plantarum* (23%). Other *Lactobacillus* species, such as *L. johnsonii* (6%), *L. oris* (3%), *L. agilis* (5%), and *L. kitasatonis* (5%), appeared less frequently. The remaining species (*L. curvatus, L. crispatus, L. fermentum,* and *L. ingluviei*) were about 1% of all isolates. The detailed data are given in Figure 1.

### 3.2 Preliminary characteristics of isolated strains: anti‐*Campylobacter* activity in vitro*,* lactic acid production, and adhesion properties

Subsequent stepwise analysis was performed using *Lactobacillus* strains isolated from fecal samples of two privately owned “back‐yard” farms, where chickens were allowed access to outdoor areas. The rationale behind this decision was based on two assumptions. First, of all these chickens were never given antibiotics as feed additives at any stage of life. It has been documented that antibiotics may influence the composition of chicken microflora for long periods (Pan & Yu, 2014; Wise & Siragusa, 2007). Second, their food, though difficult to describe in detail, was more diverse than that used on commercial poultry farms.

The preliminary experiments evaluated the inhibitory effect of the *Lactobacillus* isolates on the growth of *Campylobacter* using spot titer assays for 64 *Lactobacillus* strains, where two *C. jejuni* strains (81176 and 12) of different origins were the target strains. *C. jejuni* 81176 is human isolate that is widely used in pathogenesis studies, and it has gone through many laboratory passages since it was first isolated (Korlath, Osterholm, Judy, Forfang, & Robinson, 1985). *C. jejuni* 12 is fresh chicken isolate obtained in our lab. First we checked whether cell‐free supernatants (CFS) of analyzed *Lactobacillus* strains displayed anti‐*Campylobacter* activity. The pH of the culture supernatants ranged from 3 to 5 (predominantly 4). We found that anti‐*Campylobacter* activity was species dependent. Members of *L. reuteri* species did not exhibit an antagonistic effect on *Campylobacter* growth. Members of *L. curvatus*,* L. ingluviei*,* L. kitasatonis*, and *L. oris* also did not inhibit *Campylobacter* growth, though these results should be treated with caution due to the small number of strains analyzed. Among the strains of *L. plantarum* and *L. salivarius* species analyzed, some differences in the size of the inhibition halos were observed (Table S1). Similar results were obtained for both *Campylobacter* strains.

Next, to find the chemical nature of the CFS component responsible for *Campylobacter* growth inhibition, the CFSs were neutralized before using them for spot titer assays. We found that none of the neutral supernatants demonstrated an antagonistic effect on *Campylobacter*, independent on the pathogen strains, suggesting that the inhibition was due to acid production.

To shed more light on the mechanism by which *Lactobacillus* isolates exert their action, the levels of lactic acid present in the supernatants of the cultures of all *Lactobacillus* strains were evaluated. In general, we found that *L. reuteri* strains produced relatively lower amount of lactic acid when compared to *L. plantarum* or *L. salivarius*. However, the supernatants of the *L. reuteri* culture reveal a pH of about 4, suggesting production of the different organic acids by *L. reuteri* isolates. Of four examined *L. johnsonii* strains, two generated high levels of lactic acid. In the case of other *Lactobacillus* species (*L. agilis*,* L. crispatus*,* L. curvatus*,* L. ingluviei*,* L. kitasatonis,* and *L. oris*), too few strains were examined to make a conclusion concerning the ability of a particular species to generate lactic acid. However, in general and similar to the *L. reuteri* strains, they produced mild level of lactic acid that did not translate into antagonistic effect against *Campylobacter*.

It is postulated that highly adhesive probiotic bacteria have the greatest beneficial effects on host health and they should, at least transiently, colonize the host gut (Bermudez‐Brito et al., 2012). The adhesion microtiter plate assay is a well‐known and simple method for a preliminary assessment of strain adhesion ability (Radziwill‐Bienkowska et al., 2014), which, as we observed in our previous studies, can correlate with its adhesiveness to biotic surfaces (Aleksandrzak‐Piekarczyk et al., 2016; Radziwill‐Bienkowska et al., 2014, 2016). In this study, we checked the adhesive properties of 64 *Lactobacillus* strains isolated from chicken gastrointestinal tracts and compared them with highly adhesive or nonadhesive *L. rhamnosus* strains (GG or LOCK 0908, respectively). The overwhelming majority of bacteria demonstrated adhesion to PS as determined by an adherence ratio higher than 2 (Table S1). The strength of this ability varied between strains and, in some cases, even exceeded the value determined for the positive control strain *L. rhamnosus* GG (adherence ratio 9.38). Among the three species most widely represented in this test (*L. plantarum*,* L. reuteri*, and *L. salivarius*), *L. plantarum* seem to have the most consistent adherence since as many as ~60% of all strains efficiently adhered to PS. However, most of them adhered moderately, and only two strains were strongly adherent. Of all *L. salivarius* strains analyzed, 58% adhered moderately or strongly. *L. reuteri* strains were slightly less adherent: half adhered either moderately (six strains) or strongly (two strains; Table S1).

On the basis of the preliminary characteristics, 11 strains were chosen for detailed examination in subsequent detailed in vitro experiments. The selection was not obvious as their activity against *Campylobacter*, lactic acid production and adhesion properties revealed a high level of diversity. The main selection criterion was the strong ability to inhibit *Campylobacter* growth. The selected strains of *L. salivarius* and *L. plantarum* differ in respect to the two other qualities. Two of *L. salivarius* strains (PA17D and PA18C) generate high levels of lactic acid but each exhibits a different level of adhesion to PS. *L. salivarius* PA14C, by contrast, is characterized by low production of lactic acid and high adhesion. The differences among the chosen *L. plantarum* strains were related to their adherence capacity to PS. *L. agilis* and *L. crispatus* were added to the list due to their strong ability to inhibit *Campylobacter* growth. *L. reuteri* strains were included in consideration of their abundance in the microbiota of “back yard” chickens, although they showed no anti‐*Campylobacter* activity.

### 3.3 Examination of the potential probiotic characteristics of selected *Lactobacillus* strains

#### 3.3.1 Lactic acid production

**Figure 2:**
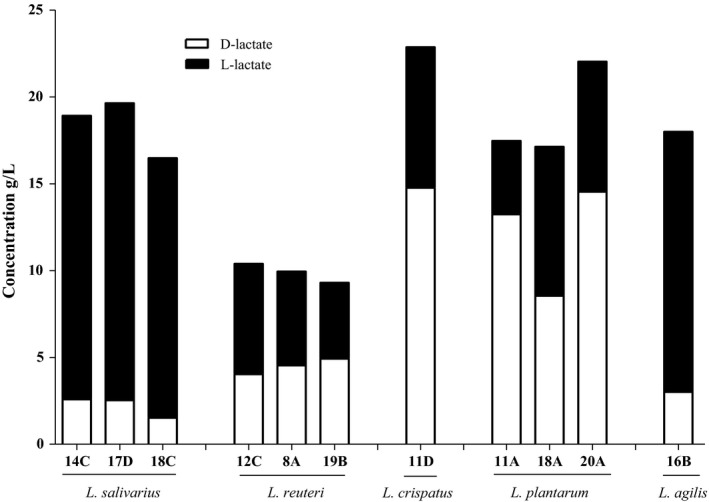
The concentrations (g/L) of d‐ and l‐lactate in the cell‐free supernatants from *Lactobacillus* culture. The concentration were measured using stereo‐specific d‐ and l‐lactate assay kits (Megazyme, Wicklow, Ireland)

First, the production of lactic acid by the lactobacilli was assessed in detail (Figure 2). The highest producers were two strains – *L. crispatus* 11D and *L. plantarum* 20A, of which the first strain produced lactic acid at a concentration of 255 mmol/L (22.9 g/L), whereas the second one was 244 mmol/L (22.1 g/L). The lowest lactic acid producers were *L. reuteri* strains – around 100 mmol/L (10 g/L). All the other strains produced 180–200 mmol/L lactic acid concentrations. The ability to produce both isoforms of lactic acid was identified for each of the *Lactobacillus* strains, and the levels of l‐ and d‐lactate enantiomers (isoforms) produced by the *Lactobacillus* strains was found to vary (Figure 2). The highest l‐lactate producers are the *L. salivarius* strains, whereas the *L. plantarum* strains demonstrated the highest levels of d‐enantiomer. The single *L. agilis* and *L. crispatus* strains were very similar in the proportions of l‐ and d‐enantiomers produced, compared to those generated by members of *L. salivarius* and *L. plantarum* species, respectively. On the other hand, *L. reuteri* strains present an intermediate group of bacteria among those tested with respect to lactate generation, since l‐ and d‐isoforms were present in almost equal concentrations.

Production of d‐lactate affects synthesis of the cell wall of the bacterial producers by incorporating the d‐lactate in place of d‐alanine, the last molecule of the pentapeptide of peptidoglycan, and thus renders the lactobacilli resistant to the antibiotic vancomycin (Ferain et al., 1996; Goffin et al., 2005). Thus resistance to vancomycin was also checked using M.I.C. Evaluator (Oxoid). We found that all but one (*L. crispatus*) of the strains were resistant to a high concentration of vancomycin (>256 μg/ml).

#### 3.3.2 Carbohydrate fermentation profiles of *Lactobacillus* sp

**Figure 3:**
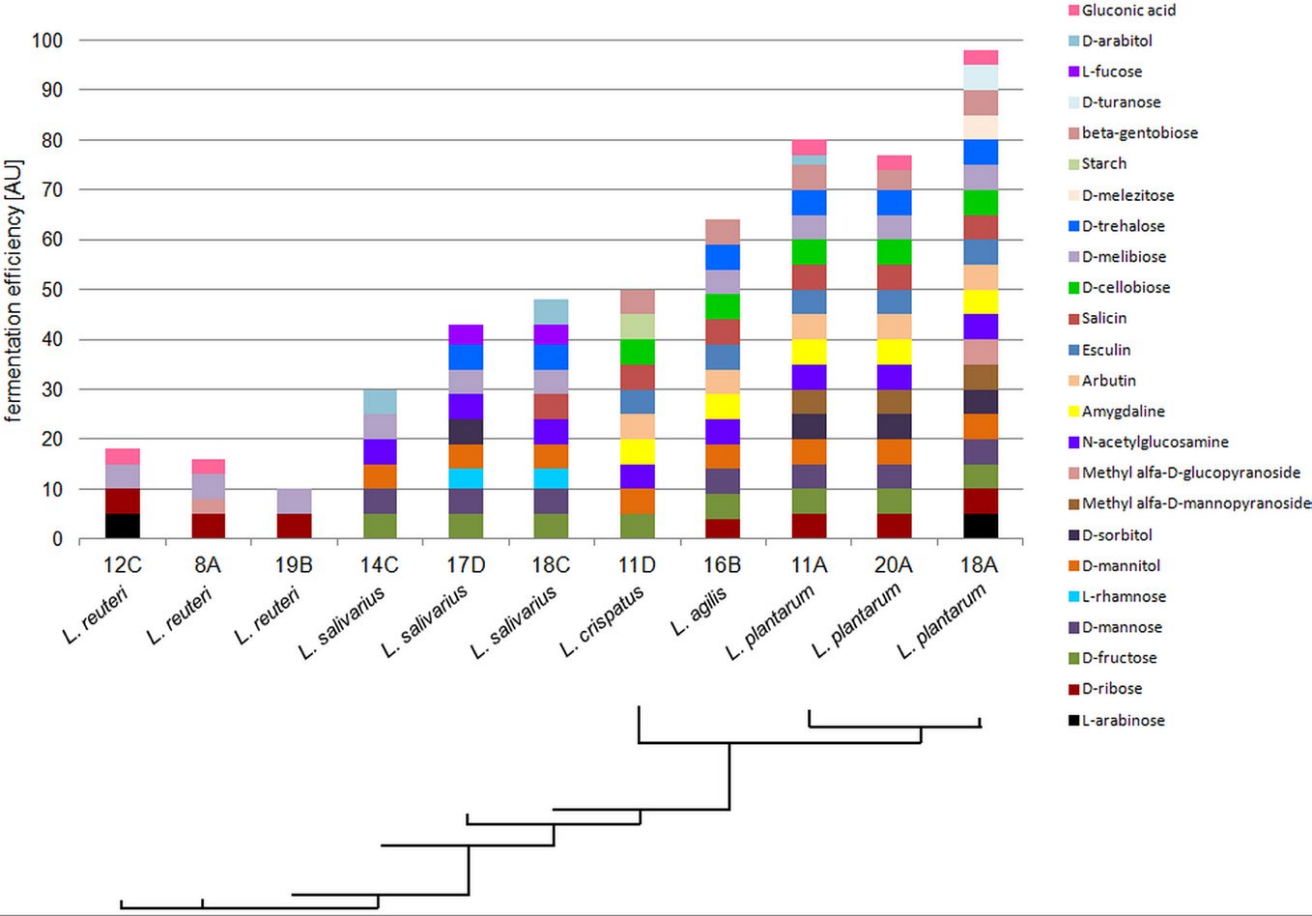
Carbon source assimilation capacities among 11 *Lactobacillus* strains. Sugar fermentation ability and efficacy is indicated by different color and size tetragons. The assimilation pattern is based on 49 selected carbon sources, excluding invariable sources. The phylogenetic relationships among the analyzed strains phenotypes are shown by a phylogenetic tree. Not used by any of the strains were: 2‐ketogluconate potassium, 5‐ketogluconate potassium, d‐adonitol, d‐arabinose, d‐fucose, d‐xylose, d‐tagatose, dulcitol, l‐xylose, erythritol, glycerol, glycogene, inositol, inuline, l‐arabitol, l‐sorbose, methyl‐α‐d‐mannopyranoside, methyl‐β‐d‐xylopyranoside, and xylitol

Utilization of many oligosaccharides from different categories appears to be an ubiquitous feature of lactobacilli (Ganzle & Follador, 2012). We analyzed the ability of selected strains (members of the *L. salivarius, plantarum, reuteri,* and *agilis* species) to grow on media containing various sugars as a carbon source, using the API test (Figure 3). The API 50 CH profiles for the strains of lactobacilli demonstrated their phenotypic diversity. *Lactobacillus* strains within a species showed identical or very similar sugar utilization profiles, but none of the three *L. salivarius* isolates shared the same phenotypic pattern. Among 49 carbohydrates present in the API 50 CH test, the assimilation of seven sugars (d‐galactose, d‐glucose, d‐lactose, d‐maltose, d‐melibiose, d‐raffinose, and d‐sucrose) was common for all analyzed *Lactobacillus* spp. (data not shown), whereas the metabolic ability and efficiency for the other 24 carbon sources (amygdaline, arbutin, d‐arabitol, β‐gentiobiose, d‐cellobiose, d‐fructose, d‐mannitol, d‐mannose, d‐melezitose, d‐ribose, d‐sorbitol, d‐trehalose, d‐turanose, esculin, gluconic acid, l‐arabinose, l‐fucose, l‐rhamnose, methyl α‐d‐glucopyranoside, methyl α‐d‐mannopyranoside, N‐acetylglucosamine, salicin, starch, and d‐ melibiose) was found to be species‐dependent. The profiles for each *Lactobacillus* strain are summarized in Figure 3. Among the species tested, all *L. plantarum* strains used the widest range of simple and more complex carbohydrates, from 22 different carbohydrates (PA11A and PA20A) up to 26 (PA18A). Among the differentially metabolized sugars (Figure 3), *L. plantarum* strains were defective in the metabolism of only two l‐form sugars (l‐rhamnose and l‐fucose).

Virtually all analyzed strains were able to metabolize maltose, the oligosaccharide from the group of α‐glucans, which are the most omnipresent sugars in the intestinal tract of grain‐eating animals (Ganzle & Follador, 2012). Also, the ability to utilize the α‐galacto‐oligosaccharides‐family (αGOS) d‐melibiose [α‐Gal‐(1 → 6)‐Glu] as well as the raffinose‐family oligosaccharide (RFO) d‐raffinose seems to be a common feature among all *Lactobacillus* strains tested. On the other hand, the metabolism of a trisaccharide, d‐melezitose (α‐Glu‐(1→3)‐β‐Fru‐(2→1)‐α‐Glu), and the disaccharides d‐trehalose [α‐Glu‐(1 → 1)‐α‐Glu] and d‐turanose (α‐Glu‐(1→3)‐α‐Fru) was species‐ or strain‐specific and restricted mainly to *L. plantarum* (Figure 3). In contrast to the ubiquitous ability to metabolize oligosaccharides, the majority of the lactobacilli were not amylolytic (Ganzle & Follador, 2012). Indeed, among 11 strains tested, only *L. crispatus* 11D showed a capacity for starch metabolism (Figure 3), indicating that this strain has a unique ability to produce an extracellular or cell‐bound α‐amylase.

In conclusion, the capacity of individual lactobacilli species for sugar metabolism differed substantially. This metabolic diversity matched with the phylogenetic diversity in the *Lactobacillus* genus (Figure 3).

#### 3.3.3 Osmotic stress resistance

The ability to resist high salt concentrations is important for probiotic bacteria, since they must survive and grow in the gastrointestinal tract where the environment has an osmolarity equivalent to 0.3 mol/L NaCl (Chowdhury, Sahu, & Das, 1996; West et al., 1996).

**Figure 4:**
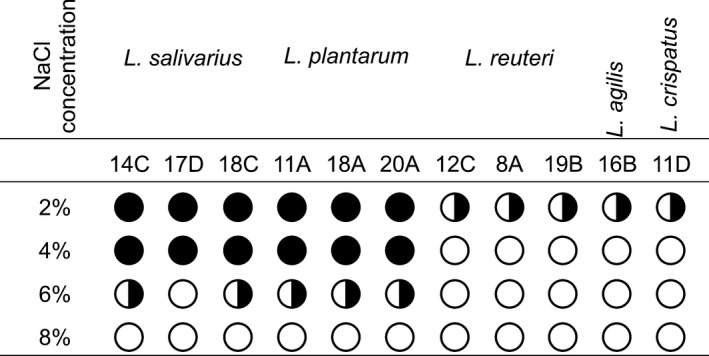
Tolerance of *Lactobacillus* isolates to different concentration of NaCl. Black circle – full resistance, B&W circle – weak resistance, white circle – lack of resistance

In this study, the isolated *Lactobacillus* spp. showed a variable capacity to survive different concentrations of NaCl. The level of bacterial osmo‐tolerance was more species‐ than strain‐dependent, but none of the isolates could grow in 1.36 mol/L NaCl. The strongest resistance (up to 1 mol/L) was found for all *L. plantarum* isolates, whereas the resistance of *L. salivarius* depended on the strain. *L. agilis*,* L. crispatus,* and *L. reuteri* strains showed the weakest resistance, with only faint tolerance to 0.3 mol/L NaCl (Figure 4).

#### 3.3.4 Tolerance to acid and bile salt

The capacity of *Lactobacillus* strains to act as probiotics is also determined by the ability to survive in the low pH of the stomach and in the high concentration of bile salt of the gastrointestinal tract (Bull, Plummer, Marchesi, & Mahenthiralingam, 2013). In our studies, we decided to check the ability of chosen strains to tolerate pH 2.5 and 0.5% bile salt (Table 2). Only one strain (*L. agilis* 16B) was unable to grow under such conditions. Other strains employed in the study, members of the species *L. plantarum* and *L. reuteri,* showed fairly good survival in low pH and in the presence of bile salt (percentage of the viability ranged from 30 to 87%). Representatives of the species *L. salivarius* showed rather high tolerance to low pH. At the same time, two of them did not grow in the presence of 0.5% bile salt (14C and 17D), and one exhibited a low percentage of survival (18C). The opposite was observed for *L. crispatus* 11D – no growth at pH 2.5 and 43.8% of viability in the presence of bile salts.

**Table 2:** The tolerance of isolated lactobacilli to low pH and bile salt

Strain	Control log_10 _CFU/ml	pH 2.5		0.5% of bile salt	
		Log_10_ CFU/ml after 6 hr	% of viability	Log_10_ CFU/ml after 6 hr	% of viability
*L. salivarius* PA14C	8.9	4.8	49.7 ± 20.4	0	0
*L. salivarius* PA17D	8.9	2.6	30.2	0	2.8 ± 5.6
*L. salivarius* PA18C	8.9	2.9	35.9 ± 31.4	3.0	32.7 ± 17.6
*L. crispatus* PA11D	7.4	0	0	3.2	43.8 ± 9.2
*L. plantarum* PA11A	7.6	4.4	58.5 ± 34.86	6.4	87.4 ± 3.5
*L. plantarum* PA18A	7.9	3.6	45.8 ± 3.7	6.6	83.1 ± 1.2
*L. plantarum* PA20A	7.6	2.3	30.1 ± 9.5	5.8	76.4 ± 7.0
*L. reuteri* PA8A	8.9	5.7	63.6 ± 3.6	4.9	55.0 ± 4.6
*L. reuteri* PA12C	8.5	3.8	44.7 ± 18.5	5.0	59.5 ± 7.4
*L. reuteri* PA19B	8.7	4.2	48.0 ± 6.6	5.0	57.7 ± 0.6
*L. agilis* PA16B	8.6	0	0	0	0

Values are the mean ± *SD* from three independent experiments.

#### 3.3.5 Oral administration of selected *Lactobacillus* strains to chickens – impact on colonization of chickens by *Campylobacter*

**Figure 5:**
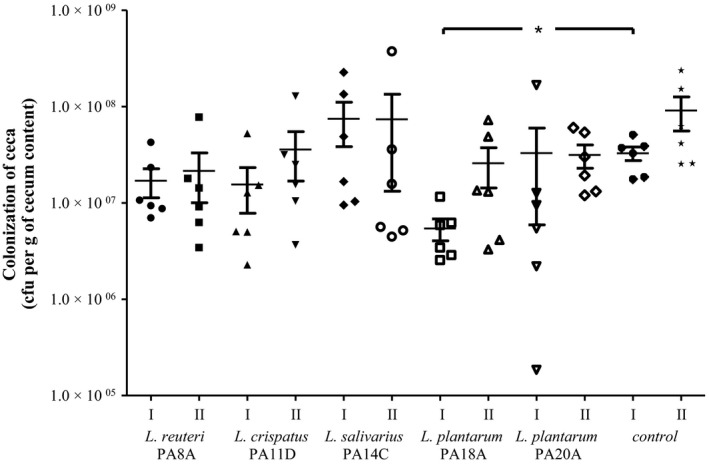
Impact of the *Lactobacillus* strains on colonization of chickens by *Campylobacter*. Chickens were orally given *Lactobacillus* strains (~10^8^ CFU) at day of hatch and 4 days post hatch. Next, birds receiving a *Campylobacter jejuni* challenge were administered *C. jejuni* 12/2 (10^4^ CFU) at day 14 post hatch. Control birds were given BSG (PBS with 0.01% gelatin). Chickens were killed at four (I) and eight (II) days post challenge. Cecal contents were serially diluted and plated onto Blood Agar for enumeration of *C. jejuni*. Bacterial recoveries represent colonization levels of six birds per time interval. The geometric mean for each group is denoted by bars. A statistical analysis was carried out using Kruskal–Wallis test. Asterisks indicate significant differences (*p *<* *.05) between analyzed groups and the control group

Of the 11 *Lactobacillus* strains characterized in detail, five were tested in vivo in chicken experiments (Table 3). The aim was to test the effectiveness of lactobacilli application to protect chicken gut against colonization by *C. jejuni*. Five groups of chickens were administered lactobacilli on day‐of‐hatch and 4 days post hatch. A group of birds inoculated with BSG (PBS with 0.1% gelatin) was used as a control. The details of the procedure are given in Materials and Methods section. At 14 days post hatch chickens were challenged with ~10^4^ CFU of *C. jejuni* 12/2 strain labeled with the pUOA18 plasmid containing a *cat* gene. Chickens were killed at 4 and 8 days post challenge and the number of *C. jejuni* present in the cecum of each chicken was determined. The mean CFU/g of cecal content observed in the untreated control group was about 3 × 10^7^ 4 days after challenge and 1 × 10^8^ CFU/g of cecal content 8 days after challenge (Figure 5). Oral inoculation of lactobacilli using strains of *L. salivarius*,* L. crispatus,* and *L. reuteri,* generally did not affect the level of chicken gut colonization by *Campylobacter*. In contrast, oral inoculation with the *L. plantarum* strains reduced the number of *Campylobacter* cells present in the chicken ceca at 4 and 8 days after challenge. The mean level of colonization in the group that received both *L. plantarum* strains 8 days after challenge was slightly reduced compared to control group (5 × 10^7^ vs. 1 × 10^8 ^CFU/g of cecal content). The reduction in *C. jejuni* cecal content due to *L. plantarum* application was apparent 4 days after challenge. In the case of *L. plantarum* PA18A, the median reduction was 1 log_10_. In the case of *L. plantarum* PA20A, the mean concentration of *C. jejuni* cells 4 days after challenge was similar to that observed for control group. However, distinct differences appeared among birds in this group. Two of six chickens were colonized at a low level (about 1 × 10^5^ and 1 × 10^6^ CFU/g of cecal content).

**Table 3:** Properties of selected *Lactobacillus* strains isolated from feces of chickens

Species	Strain	Anti‐*Campylobacter* activity	Adhesion to bare polystyrene (adherence ratio)^a^	l‐lactate (g/L)^a^	d‐lactate (g/L)^a^	% of viability at pH 2.5^a^	% of viability in the presence of 0.5% bile salt
*L. salivarius*	PA14C	+	7.80 ± 1.34	16.4 ± 0.44	2.6 ± 0.11	49.7 ± 20.4	0
	PA17D	+	6.22 ± 1.11	17.1 ± 0.40	2.5 ± 0.53	30.2	2.8 ± 5.6
	PA18C	+	3.50 ± 0.31	14.9 ± 1.03	1.5 ± 0.14	35.9 ± 31.4	32.7 ± 17.6
*L. reuteri*	PA12C	−	3.62 ± 0.41	6.4 ± 0.45	4.0 ± 0.28	44.7 ± 18.5	59.5 ± 7.4
	PA8A	−	3.66 ± 1.00	5.4 ± 0.19	4.5 ± 0.35	63.6 ± 3.6	55.0 ± 4.6
	PA19B	−	2.79 ± 0.27	4.4 ± 0.98	4.9 ± 1.19	48.0 ± 6.6	57.7 ± 0.6
*L. crispatus*	PA11D	+	3.46 ± 1.68	8.1 ± 1.09	14.8 ± 1.94	0	43.8 ± 9.2
*L. plantarum*	PA11A	+	11.24 ± 0.54	4.2 ± 0.46	13.2 ± 1.65	58.5 ± 34.86	87.4 ± 3.5
	PA18A	+	3.48 ± 0.26	8.6 ± 0.68	8.6 ± 1.04	45.8 ± 3.7	83.1 ± 1.2
	PA20A	+	1.10 ± 0.32	7.5 ± 0.66	14.6 ± 0.83	30.1 ± 9.5	76.4 ± 7.0
*L. agilis*	PA16B	+	2.20 ± 0.33	15.0 ± 1.21	3.0 ± 0.45	0	0

The *Campylobacter jejuni* inhibition assay was performed on Blood Agar plates containing 5% horse blood (Oxoid) inoculated with 100 μl of *Campylobacter* cultures. *Campylobacter* growth inhibition by supernatant of *Lactobacillus* strains is marked as +. Bacterial adhesion was determined using the technique described by Radziwill‐Bienkowska et al. (2014) in three independent experiments. Bacteria were characterized as strongly adherent (*A* ≥ 6), moderately adherent (6 > *A* ≥ 3), weakly adherent (3 > *A* > 2), and nonadherent (*A* ≤ 2). The adherence ratio for strain *L. rhamnosus* GG (positive control) was 9.38 and the ratio for nonadhesive *L. rhamnosus* LOCK 0908 (negative control) was 1.05.

The concentrations (g/L) of d‐ and l‐lactate in the cell‐free supernatants were measured using stereo‐specific d‐ and l‐lactate assay kits (Megazyme, Wicklow, Ireland).

a Values are the mean ± *SD* from three independent experiments.

## 4 DISCUSSION

A wide array of carriers, both living and nonliving, has been developed to deliver heterologous antigens to the immune system of humans or animals. Among live antigen delivery systems, live attenuated bacterial vectors have been the subject of the most thorough investigations (Curtiss, 2015). LAB bacteria have received considerable attention as biotherapeutic and immunoprophylactic agents due to their GRAS status and because members of this group of microorganisms are autochthonous residents in the human or animal gastrointestinal tract (Bermudez‐Humaran, Kharrat, Chatel, & Langella, 2011; Daniel, Roussel, Kleerebezem, & Pot, 2011; Foligne, Daniel, & Pot, 2013; Wyszynska, Kobierecka, Bardowski, & Jagusztyn‐Krynicka, 2015).

We have shown that in ovo chicken immunization with liposomes containing *Campylobacter* hybrid protein rCjaAD results in a moderate protection against colonization (Kobierecka, Wyszynska, et al., 2016). The same hybrid protein expressed on the surface of *L. lactis* also exerted a moderately positive effect, when used for chicken vaccination (Kobierecka, Olech, et al., 2016). We postulate that the efficacy of in ovo immunization with nonliving carriers may be increased by a boost and immunization after hatching with live vectors carrying *Campylobacter* antigens known to induce a mucosal immune response. The main drawback of the *L. lactis* delivery vehicle is the lack of long‐lasting colonization of the chick digestive tract. Using *Lactobacillus* spp. that can consistently colonize, or at least persist for an extended period in the bird intestine and display probiotic properties, should result in more efficient induction of the immune response than that exerted by *L. lactis. Lactobacillus* strains may be used to decrease the number of *Campylobacter* colonizing the chicken digestive tract, but at the same time, they can be employed as a vector to deliver *Campylobacter* immunodominant proteins to the bird immune system. However, members of *Lactobacillus* spp. constitute an extremely diverse group as regards of their physiological attributes. Thus the main goal of the present work was to identify and characterize *Lactobacillus* strains isolated from chicken breeds in Poland. First, we compared the composition of *Lactobacillus* genera colonizing the digestive tract of chickens reared under different conditions. Various species of *Lactobacillus* genus, such as *L. plantarum, L. agilis, L. brevis, L. reuteri, L. salivarius, L. fermentum,* and *L. crispatus,* have been isolated from chicken digestive tracts (Abbas Hilmi, Surakka, Apajalahti, & Saris, 2007; Hammons et al., 2010; Lin et al., 2007; Noohi, Ebrahimipour, Rohani, Talebi, & Pourshafie, 2014). However, studies performed in various countries reported different species as being dominant. The study conducted by Hammons et al. (2010) indicated that the chicken feeding affects the type of *L. agilis* strains colonizing the birds. Due to enormously discrepant data concerning lactobacilli colonizing chicken gut, we decided to examine lactobacilli strains isolated from birds reared in Poland, as they probably have evolved together with their hosts. The results of this study showed that although the *L. salivarius* was a dominant species in both “back‐yard” and commercial chickens, the species distribution among the examined *Lactobacillus* strains isolated from fecal samples of “back‐yard” chickens was more diverse than that among isolates from commercially reared birds. The more diversified diet of privately owned birds may influence the composition of their microbial community and can have an impact on which bacterial strains become dominant in the bird intestine. Also it should be noted that “back‐yard” chickens, in contrast to commercially reared birds, have contact with microorganisms colonizing other animals or present in the environment.

*Lactobacillus plantarum* species were only isolated from intestines of “back yards” chickens. Analysis of the sugars utilized by each strain showed that three *L. plantarum* strains were able to utilize an enormously wide spectrum of sugars, from 22 different carbohydrates up to 26. Among all the sugars analyzed, the *L. plantarum* strains were unable to metabolize only two l‐form sugars (l‐rhamnose and l‐fucose) and starch. This fact suggests the high versatility of this species. Indeed, *L. plantarum* is commonly found in many different ecological niches, such as vegetables, meat, fish, dairy products as well as in the gastrointestinal tracts (Siezen & van Hylckama Vlieg, 2011). This broad repertoire of sugar metabolism corresponds with the more than 3 Mb *L. plantarum* genome, which is among the largest *Lactobacillus* genomes reported to date (http://www.ncbi.nlm.nih.gov/genome/). Similar to *L. plantarum*, a slightly narrower carbohydrate utilization profile was observed for *L. agilis*. In contrast, the observed spectrum of metabolic activity of all the *L. salivarius* and *L. reuteri* isolates was low and, among the differentially metabolized sugars, limited to only a few carbon sources. Neither *L. salivarius* nor *L. reuteri* was able to metabolize any of the β‐glucosides, and additionally, all *L. reuteri* strains were defective in metabolism of α‐glucosides and more simple sugars, such as d‐arabitol, d‐fructose, d‐mannitol, d‐mannose, l‐rhamnose, and d‐sorbitol metabolism. The significant reduction in the range of sugars metabolized by *L. salivarius* and *L. reuteri*, as compared to *L. plantarum*, may result from the considerably smaller genome size of both species (http://www.ncbi.nlm.nih.gov/genome/), and that genome size may be subject to an ongoing process of degradation for niche adaptation, as was found in the case of *L. salivarius* (Raftis, Salvetti, Torriani, Felis, & O'Toole, 2011). *L. salivarius* and *L. reuteri* are reported to be highly specialized, and their primary habitats are limited to niches such as human and animal gastrointestinal tracts (Casas & Dobrogosz, 2000; Raftis et al., 2011).

Acid and bile tolerance and the ability to overcome high concentration of NaCl are essential natural attributes of probiotic strains since they have to survive in the conditions present in the intestinal tract (stomach and small intestine) of their host. Thus, we checked whether the selected strains display these properties. In this study, the isolated *Lactobacillus* sp. showed a variable capacity to survive in different concentrations of NaCl. According to Mohd Adnan and Tan (2007), high osmotolerance could be a desirable feature for potentially commercial LAB strains, because lactic acid production, would result in pumping alkali outside to prevent an excessive reduction in pH, and converting the free acid to its salt form, finally elevating the osmotic pressure on the bacterial cells (Mohd Adnan & Tan, 2007). Our results suggest that all the analyzed strains could resist the NaCl osmolarity of the animal GI tract, but only *L. plantarum* and some *L. salivarius* strains could resist technological processing, as they were the most tolerant to high NaCl concentrations that can be present in pelleted or dried animal food. Moreover, it has been reported that *Lactobacillus* strains adapted to NaCl exhibited up to 16‐fold higher tolerance to lethal temperatures used during spray drying technological process (Desmond, Stanton, Fitzgerald, Collins, & Ross, 2001).

One possible mechanism by which lactobacilli restrict the growth of pathogenic microorganisms is their ability to produce inhibitory metabolites such as organic acids. Lactic acid is widely involved in various branches of industry, for example, food and feed production, medical and pharmaceutical use, and also in cosmetics. The biologically active isoform of lactate is l‐lactate (Martinez et al., 2013), which unlike d‐lactate, can be metabolized by humans. Lactic acid impairs growth of various microorganisms, including pathogenic and spoilage bacteria (Neal‐McKinney et al., 2012).

Members of the *Lactobacillus* genus are l‐ and d‐lactate producers, though the ratio of two isomers differs considerably and is recognized as species dependent (Manome, Okada, Uchimura, & Komagata, 1998). Our results indicate that this feature of lactobacilli may be not only species‐ but also strain‐dependent. The *L. crispatus* strain characterized by Neal‐McKinney et al. (2012) produces similar levels of two lactate isomers, whereas *L. crispatus* analyzed in this work produces mainly d‐lactate. Although high l‐lactate concentrations were observed only for the *L. salivarius* and *L. agilis* strains, strains with high d‐lactate levels also impaired growth of the *Campylobacter* pathogen. It is thus possible, that a certain level of total lactate concentration (both l‐ and d‐isoforms) is important to hamper the growth of pathogenic bacteria. Production of d‐lactate, mediated by either d‐lactate dehydrogenase or l‐lactate racemase, affects synthesis of the cell wall of the producer bacteria that incorporate it in place of d‐alanine, the last molecule of the pentapeptide of peptidoglycan, which renders the lactobacilli resistant to the high concentrations of antibiotic vancomycin (Ferain et al., 1996; Goffin et al., 2005). This phenotype is seen mostly in *L. plantarum* and some other heterofermentative lactobacilli. Vancomycin resistance of homofermentative *L. salivarius* species is controversial and potentially is strain‐dependent (Ammor, Florez, & Mayo, 2007; Ammor et al., 2008; EFSA, 2012). Our observations corroborate these data, since all the strains tested were found to be resistant to vancomycin (intrinsic resistance, nonspecific, nontransferable). The only strain sensitive to vancomycin was *L. crispatus,* which similar to *L. plantarum* produces high levels of d‐lactate and belongs to the facultatively heterofermentative group of lactobacilli. Explanation of this vancomycin sensitivity requires more research, as the mechanism that determines vancomycin resistance was studied in detail only in *L. plantarum* (Goffin et al., 2005).

Anti‐*Campylobacter* activity expressed in in vitro experiments has been documented for some *Lactobacillus* strains of different origins (Ghareeb et al., 2012; Robyn et al., 2012; Santini et al., 2010). Although many *Lactobacillus* strains have been examined for their anti‐*Campylobacter* activity in vitro*,* only limited studies to date have been undertaken to confirm this effect in vivo. Our knowledge about interaction among constituents of intestinal microbiota is rather limited. Thus, the data arising from in vitro experiments, although they provide useful information about strain physiology, have to be treated with caution and only as indication for planning further experiments. To evaluate anti‐*Campylobacter* activities in vivo*,* potential probiotic *Lactobacillus* species have been administered as mono‐ or multi‐species feed additives. The studies have differed in their experimental designs and the data obtained through in vitro examination were not always consistent with those provided by animal studies (Robyn, Rasschaert, Hermans, Pasmans, & Heyndrickx, 2013; Robyn et al., 2012; Santini et al., 2010). The main differences among in vivo experiments concern the length of time of formula administration and *C. jejuni* strains used (Neal‐McKinney et al., 2012; Nishiyama et al., 2014; Santini et al., 2010). The procedures most often used daily administration of the probiotic formula. Although most of *Lactobacillus* strains only transiently colonize chicken gastrointestinal chicken tracts, three *Lactobacillus* strains (*L. agilis*,* L. crispatus,* and *L. vaginalis*) that were able to persist in the chicken intestine for the entire production period after a single inoculation (day 0) have been isolated (Spivey et al., 2014; Stephenson, Moore, & Allison, 2010). Generally the clearest efficacy was achieved by daily applying a multispecies probiotic product (Poultry Star sol) (Ghareeb et al., 2012; Guyard‐Nicodeme et al., 2016). In the present work, we employed a schema of chicken experiments identical as that used by Neal‐McKinney et al. (2012), but some elements differed, mainly the *C. jejuni* strain used for challenge and the *Lactobacillus* strains administered. The one common *Lactobacillus* strain used by us and Neal‐McKinney et al. was a member of the *L. crispatus* species, but the two strains appear to differ in terms of their physiology, as based on type and amount of lactate production. Of five species tested in this work, lactobacilli strains that are members of *L. plantarum* appeared to be the most effective. Other strains did not display anti‐*Campylobacter* activity, though it is not known whether a more frequent and longer application period would improve their efficacy.

Overall, the present data show that *L. plantarum* strains isolated from digestive tracts of chicken bred in Poland displayed some probiotic attributes in vitro and were able to decrease the level of bird gut colonization by *C. jejuni* strain. However, further experiments are needed to understand details concerning their interaction with *C. jejuni,* as well as with the host, which should help to improve the schema of its application. Additionally, recent experiments in our lab show that *L. plantarum* strains are susceptible to genetic manipulation, so they may be used as potential delivery vectors for *C. jejuni* antigens.

## CONFLICT OF INTEREST

None declared.
