# Supplementary figures and images for: In vitro characteristics of Lactobacillus spp. strains isolated from the chicken digestive tract and their role in the inhibition of Campylobacter colonization
Source: Microbiologyopen. 2017 Jul 24;6(5):e00512. doi: 10.1002/mbo3.512 (PMC5635155; doi:10.1002/mbo3.512)

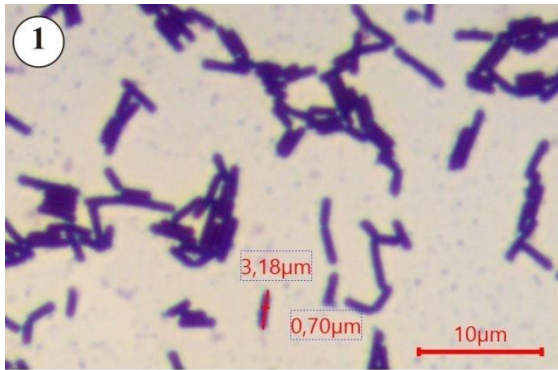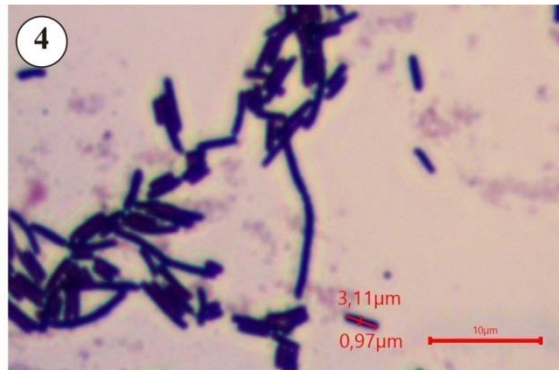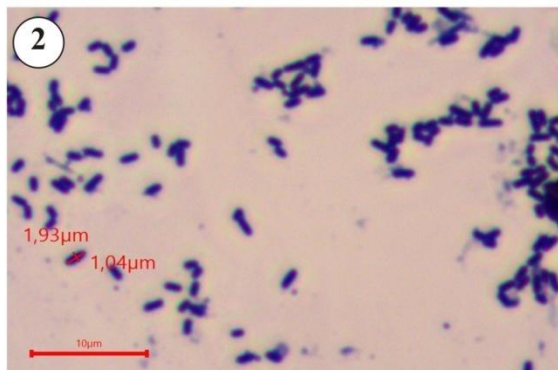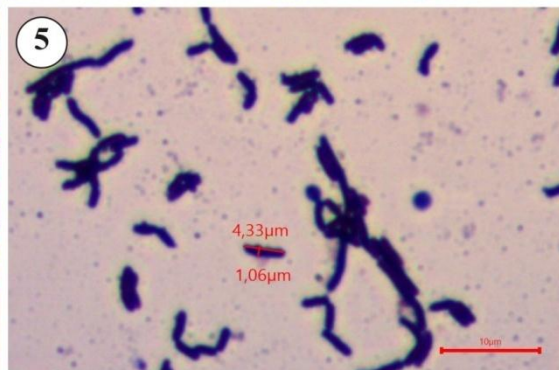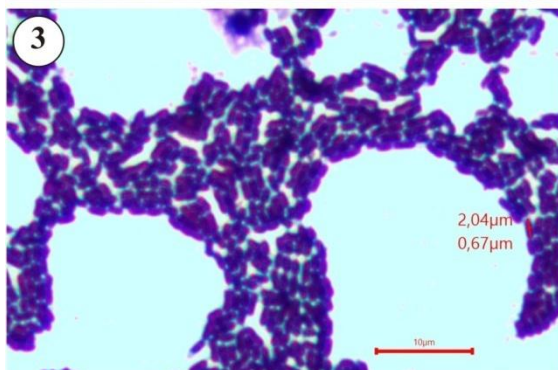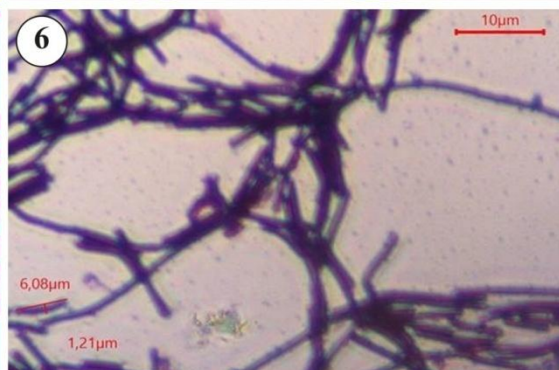

Supplement: Supplementary file 1 [file MBO3-6-na-s001.pdf]
