# Supplementary material for: In vitro characteristics of Lactobacillus spp. strains isolated from the chicken digestive tract and their role in the inhibition of Campylobacter colonization
Source: Microbiologyopen. 2017 Jul 24;6(5):e00512. doi: 10.1002/mbo3.512 (PMC5635155; doi:10.1002/mbo3.512)
Supplement: Supplementary file 2 [file MBO3-6-na-s002.docx]

| Species | Isolated strain | Anti-  *Campylobacter* activity *in vitro* | Adhesion to bare polystyrene  (adherence ratio) | Lactic acid production | |
| --- | --- | --- | --- | --- | --- |
|  |  |  |  | L | D |
| *L. salivarius* | 2I | + | + | +++! | + |
| *L. salivarius* | 2B | + | +++ | ++ | + |
| *L. salivarius* | 3C | + | ++ | +++! | + |
| *L. salivarius* | 3F | nd | ++ | ++ | ++ |
| *L. salivarius* | 5F | +/- | +++ | + | nd |
| *L. salivarius* | 6H | +! | nd | +++! | + |
| *L. salivarius* | 7A | - | +++ | +++! | + |
| *L. salivarius* | 8D | + | + | +++ | + |
| *L. salivarius* | 8F | + | - | +++ | + |
| *L. salivarius* | 9B | nd | - | +++ | + |
| *L. salivarius* | 9F | + | + | +++! | + |
| *L. salivarius* | 14C | +! | +++ | + | + |
| *L. salivarius* | 15C | +! | +++ | +++! | + |
| *L. salivarius* | 17A | + | ++ | +++! | + |
| *L. salivarius* | 17D | +! | +++ | +++! | + |
| *L. salivarius* | 18B | +! | - | ++ | ++ |
| *L. salivarius* | 18C | +! | ++ | +++! | + |
| *L. salivarius* | 15B | + | ++ | +++ | + |
| *L. reuteri* | 1E | - | - | ++ | + |
| *L. reuteri* | 2A | - | ++ | ++ | + |
| *L. reuteri* | 4A | nd | nd | ++ | + |
| *L. reuteri* | 4D | - | - | ++ | + |
| *L. reuteri* | 5B | - | + | ++ | + |
| *L. reuteri* | 5C | - | ++ | ++ | + |
| *L. reuteri* | 8A | - | ++ | ++ | + |
| *L. reuteri* | 8E | - | ++ | ++ | + |
| *L. reuteri* | 9E | - | - | ++ | + |
| *L. reuteri* | 10A | - | ++ | ++ | + |
| *L. reuteri* | 10D | - | + | ++ | ++ |
| *L. reuteri* | 12B | - | +++ | ++ | + |
| *L. reuteri* | 12C | - | ++ | + | + |
| *L. reuteri* | 14A | - | +++ | ++ | + |
| *L. reuteri* | 15D | nd | + | ++ | + |
| *L. reuteri* | 19B | - | + | ++ | + |
| *L. plantarum* | 3A | +! | ++ | +++ | +++ |
| *L. plantarum* | 6A | - | ++ | ++ | ++ |
| *L. plantarum* | 7F | +/- | nd | ++ | ++ |
| *L. plantarum* | 10B | +! | ++ | +++ | ++ |
| *L. plantarum* | 11A | +! | +++ | ++ | +++ |
| *L. plantarum* | 11E | +! | +++ | +++ | +++ |
| *L. plantarum* | 13A | +! | ++ | ++ | +++ |
| *L. plantarum* | 13B | +! | - | ++ | ++ |
| *L. plantarum* | 13C | + | - | ++ | +++ |
| *L. plantarum* | 17E | +! | - | ++ | +++ |
| *L. plantarum* | 18A | +! | ++ | ++ | ++ |
| *L. plantarum* | 19C | +! | ++ | +++ | ++ |
| *L. plantarum* | 20A | + | - | ++ | +++ |
| *L. plantarum* | 20C | +/- | - | +++ | ++ |
| *L. agilis* | 1A | +/- | - | ++ | ++ |
| *L. agilis* | 16A | +/- | ++ | + | ++ |
| *L. agilis* | 16B | +! | + | +++! | + |
| *L. kitasatonis* | 6I | +/- | - | + | ++ |
| *L. kitasatonis* | 12A | - | - | ++ | ++ |
| *L. kitasatonis* | 19A | +/- | ++ | ++ | ++ |
| *L. johnsonii* | 3B | +! | ++ | ++ | +++ |
| *L. johnsonii* | 5D | +/- | - | ++ | +++ |
| *L. johnsonii* | 11C | +/- | ++ | + | + |
| *L. johnsonii* | 17C | +/- | ++ | + | ++ |
| *L. fermentum* | 1G | - | ++ | + | + |
| *L. curvatus* | 16C | - | ++ | ++ | ++ |
| *L. crispatus* | 11D | +! | ++ | ++ | ++ |
| *L. oris* | 14B | - | nd | + | + |
| *L. oris* | 15E | - | ++ | + | + |
| *L. ingluviei* | 8G | - | - | ++ | ++ |
